# *In Vitro* Drug Absorption Enhancement Effects of *Aloe vera* and *Aloe ferox*

**DOI:** 10.3797/scipharm.1202-10

**Published:** 2012-04-01

**Authors:** Catharina Beneke, Alvaro Viljoen, Josias Hamman

**Affiliations:** 1 Department of Pharmaceutical Sciences, Tshwane University of Technology, Private bag X680, Pretoria, 0001, South Africa; 2 Unit for Drug Research and Development, North-West University, Private Bag X600, Potchefstroom, 2520, South Africa

**Keywords:** *Aloe ferox*, *Aloe vera*, Drug absorption enhancement, Transepithelial electrical resistance, *In vitro* transport

## Abstract

The effect of whole leaf and gel materials from two aloe species (*Aloe vera* and *A. ferox*) was compared with that of the precipitated polysaccharides from these aloe materials on the transepithelial electrical resistance (TEER) as well as transport of a model compound (atenolol) in the apical-to-basolateral direction across rat intestinal tissue. All the aloe leaf materials and precipitated polysaccharides had a statistically significant effect of lowering the TEER (P < 0.05) compared to the control group, which indicates their ability to open tight junctions between adjacent epithelial cells. In contrast to the expectation from the TEER results, only the precipitated polysaccharides from dehydrated *A. vera* gel (Daltonmax 700^®^) had a statistically significant effect of enhancing the transport of atenolol (P < 0.05). These *in vitro* results therefore indicate that *A. vera* gel polysaccharides have potential as drug absorption enhancing agents in novel pharmaceutical drug delivery systems.

## Introduction

One function of epithelial cells is to maintain distinct compartments within the body and also to act as barriers to separate the body from the external environment. Although molecules can cross the intestinal epithelium by three main pathways, namely transcellular passive diffusion, paracellular passive diffusion and carrier-mediated transport, many useful drugs exhibit poor absorption after oral administration [1]. Poor drug absorption across the intestinal epithelium is in many cases attributed to unfavourable physico-chemical properties of the drug molecule such as hydrophilicity and a large molecular weight. The intestinal absorption of these drugs can be increased by different techniques such as co-administration of absorption enhancing agents. Absorption enhancing agents may facilitate the absorption of poorly absorbable drugs by different mechanisms such as opening of tight junctions or changing the membrane structure or targeting transporter proteins. Unfortunately, damage to the mucosal epithelium is a major problem with drug absorption enhancing agents [2]. Evidence that certain drug absorption enhancing agents can increase intestinal drug absorption in a reversible way without causing damage or toxic effects have ignited renewed interest in finding safe and effective drug absorption enhancers to increase drug bioavailability [3]. Tight junctions between epithelial cells are dynamic structures that can be modulated by certain chemicals in such a way to enlarge the pores or fenestrae and thereby allow paracellular passage of hydrophilic macromolecules. This approach to drug absorption enhancement has the additional advantage of avoiding enzymatic degradation of susceptible molecules. Compounds that selectively open the intestinal epithelial tight junctions, referred to as paracellular permeability enhancers, have shown potential as novel excipients in advanced drug delivery systems [1, 4].

*Aloe vera* L. is one of approximately 420 aloe species belonging to the Xanthorrhoeaceae [5]. Aloe gel is the colourless fraction contained in the inner part of the fresh leaves [6]. This inner gel is composed of large thin-walled parenchyma cells filled with the gel composed of mono- and polysaccharides [7]. The polysaccharides in *A. vera* gel consist mainly of linear chains of glucose and mannose molecules with considerably more mannose present than glucose. Acemannan (or aloverose) is a β-(1,4)-linked galactomannan with acetylated mannose residues [8]. Some of the pharmacological activities of acemannan include antiviral effects, wound healing acceleration, anti-cancer, activation of macrophages and stimulation of T cells [8–11]. Another high molecular weight polysaccharide isolated from aloe gel is aloeride. A smaller form of highly acetylated polysaccharide known as modified aloe polysaccharide was isolated from cellulose-treated aloe gel [12].

*Aloe ferox* has been harvested mainly for its exudates or sap for almost 250 years. The plants are widely distributed in South Africa and are largely concentrated in the Eastern and Western Cape Provinces [13]. Monosaccharides found in *A. ferox* gel include glucose, arabinose, galactose, rhamnose and xylose. It is speculated that there are three chemotypes of *A. ferox* species, namely glucose only, galactose-glucose (1:1) and galactose-glucose (1:2). Xylose is a minor sugar found in this aloe species with only trace amounts sporadically present [14].

An *in vivo* study on *A. vera* gel and whole leaf extract showed the enhancement of the bioavailability of vitamins C and E in humans [15], while an *in vitro* study showed that *A. vera* gel and whole leaf materials were able to significantly reduce transepithelial electrical resistance (TEER) of Caco-2 cell monolayers and also significantly enhanced the transport of insulin across this cell culture model. From this previously reported study it was concluded that the transport enhancement effect of *A. vera* leaf materials is probably due to the opening of tight junctions to allow paracellular transport [16]. This study aimed to determine the contribution of polysaccharides in the aloe materials on their drug absorption enhancing properties by comparing the effect of precipitated polysaccharides to that of aloe leaf gel and whole leaf extracts on the TEER and transport of atenolol as a model compound across excised rat intestinal tissue.

Atenolol is a highly hydrophilic β_1_ antagonist that is used in the treatment of hypertension, which is incompletely absorbed after oral administration, and only about 50 % of the administered dose reaches the systemic circulation. Due to its relatively low membrane permeability, it has been used before as a model compound for *in vitro* drug absorption enhancing investigations [17].

## Results and Discussion

### ^1^H-NMR fingerprinting of materials

**Fig. 1.**
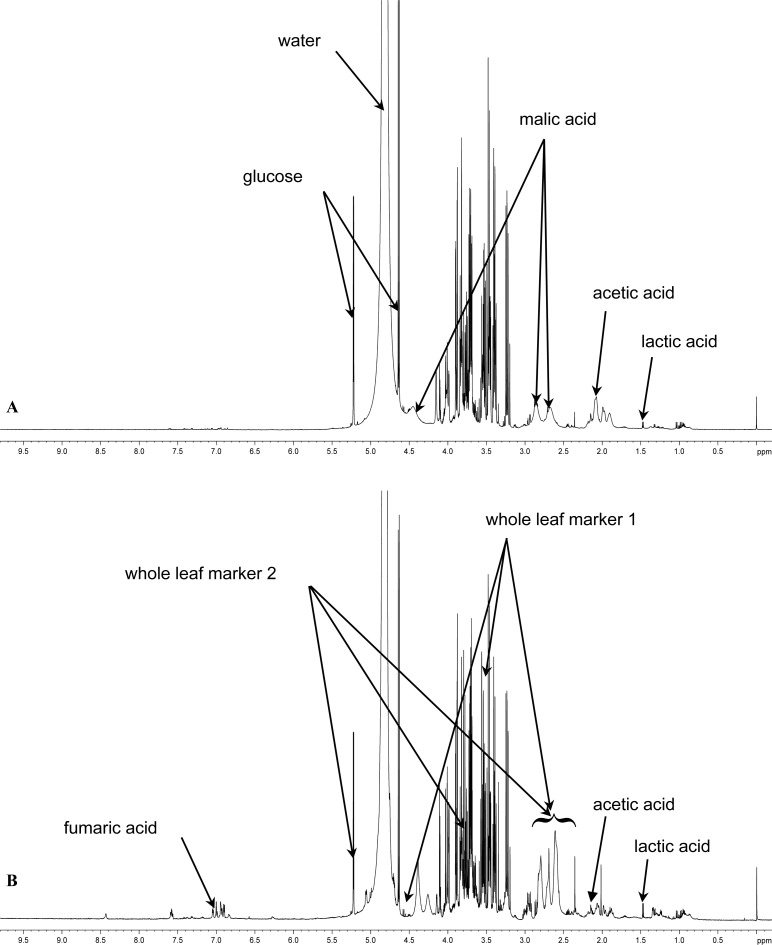
^1^H-NMR spectra of (A) *Aloe ferox* gel material and (B) *Aloe ferox* whole leaf material

The composition of the different *A. vera* materials and precipitated polysaccharides investigated in this study in terms of marker molecule quantities as determined from their respective ^1^H-NMR spectra are summarised in Table 1. The ^1^H-NMR spectra of the *A. ferox* gel and *A. ferox* whole leaf extract are shown in Figure 1a and 1b, respectively.

All the *A. vera* leaf materials and their precipitated polysaccharides investigated in this study contained aloverose (or acetylated polymannose or acemannan), which is a marker molecule for identification of fresh *A. vera* leave material [16]. The *A. vera* whole leaf extract materials also contained whole leaf marker (WLM), which confirmed their identity. On the other hand, the *A. ferox* gel and whole leaf materials did not contain aloverose, but some of the other marker molecules characteristic of aloe leaf materials were detected as indicated on the spectra in Figure 1A and 1B.

### Transepithelial electrical resistance of excised rat intestinal tissue

**Fig. 2.**
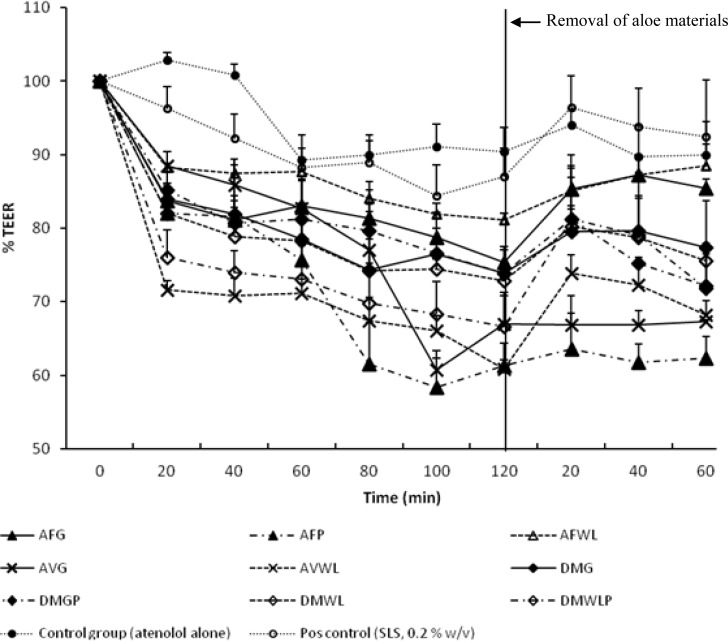
Percentage transepithelial electrical resistance (TEER) of excised rat intestinal tissue plotted as a function of time for the control and experimental groups. AVG = *Aloe vera* gel; AVWL = *Aloe vera* whole leaf; AFG = *Aloe ferox* gel; AFWL = *Aloe ferox* whole leaf; AFP = *Aloe ferox* polysaccharide; DMG = Daltonmax700^®^ gel; DMWL = Daltonmax700^®^ whole leaf; DMGP = Daltonmax700^®^ gel precipitated polysaccharides; DMWLP = Daltonmax700^®^ whole leaf precipitated polysaccharides.

The effect of the aloe materials investigated in this study at a concentration of 2% w/v on the TEER of excised rat intestinal tissue is presented in Figure 2.

TEER is a measure of tight junction integrity between adjacent intestinal epithelial cells [18]. If the size of the openings of the tight junctions increases in the presence of a paracellular permeability enhancer, the TEER of the intestinal epithelium will be reduced because of the increasing flow of ions through the opened tight junctions and intercellular spaces [1]. All the aloe materials investigated in this study lowered the TEER of the excised rat intestinal tissue statistically significantly (P < 0.05) compared to the control group (atenolol alone). The effect of all the aloe materials on the TEER of the excised rat intestinal tissue was furthermore greater than that of the positive control group (SLS 0.2% w/v), which exhibited a reduction in TEER of only 13.02%.

Some of the precipitated polysaccharides (i.e. DMWLP and AFP) had higher effects on the TEER than their corresponding gel and whole leaf materials, possibly due to higher concentrations of the polysaccharides. This may indicate that the polysaccharides in the aloe materials are responsible for or contribute to a large extent to the effect on the TEER of the excised rat intestinal tissue. This reduction in TEER of the excised rat intestinal tissue by the aloe materials indicates their ability to open the tight junctions between epithelial cells, which indicates the potential of these materials to enhance drug transport across intestinal tissues [19]. Furthermore, the reduction effect on the TEER of the excised rat intestinal tissue by the aloe materials is in line with previous findings where *A. vera* gel and whole leaf materials were able to reduce the TEER of Caco-2 cell monolayers statistically significantly [16].

After removal of the aloe materials from the apical side of the excised rat intestinal tissue, the TEER started to return towards the initial value but only partial recovery of the TEER was observed for all the aloe materials over a period of 60 min. Since a full recovery of TEER was obtained in the positive control group (SLS, 0.2% w/v), the partial return of TEER towards the initial value for the test groups containing aloe materials may be explained by the higher viscosity of these solutions. Due to their higher viscosity it was difficult to remove these solutions completely without damaging the excised rat intestinal tissue membranes as explained before for high molecular weight polysaccharides such as chitosan and N-trimethyl chitosan chloride [20].

### Atenolol transport across excised rat intestinal tissue

The calculated apparent permeability coefficient (P_app_) values for atenolol transport in the absence (control group) and presence of sodium lauryl sulphate (positive control group) and the aloe materials are shown in Table 2.

According to the P_app_ values (Table 2), the precipitated polysaccharides from the Daltonmax 700^®^ dehydrated *A. vera* gel (DMGP) was able to enhance the transport of atenolol statistically significantly (P < 0.05) across excised rat intestinal tissue at concentrations of 2.0% w/v compared to the control group (atenolol alone). The effect of these precipitated polysaccharides on atenolol transport was similar to that obtained with the positive control group (SLS at 0.2% w/v) with absorption enhancement ratio values (R) of 1.84 for both. The transport of atenolol in the presence of the precipitated polysaccharides from the Daltonmax 700^®^ *A. vera* whole leaf extract was substantially higher than that of the control group (atenolol alone), but it was not statistically significant. *A. vera* gel increased the transport of atenolol only to a relatively low extent compared to that of the control group (atenolol alone), which was also not statistically significantly. In contrast to the TEER results, some of the aloe gel and whole leaf materials including Daltonmax700^®^ *A. vera* dehydrated gel and whole leaf extract (DMG and DMWL) as well as *A*. ferox gel and whole leaf extract (AFG and AFWL) and precipitated polysaccharide (AFP) decreased the transport of atenolol slightly across the excised rat intestinal tissue when compared to the control group. The slight reduction in atenolol transport by some of the aloe materials may be explained by mechanisms such as increased viscosity of the drug solution through which the drug has to diffuse, potential blocking of the intercellular spaces by components of the aloe materials and/or potential interaction of certain components of the aloe materials with atenolol to form complexes. However, these mechanisms need to be investigated further in order to be conclusive regarding this phenomenon.

The significant and pronounced increase in atenolol transport by the precipitated polysaccharides from Daltonmax700^®^ gel and whole leaf extract (DMGP and DMWLP) materials respectively may indicate that the polysaccharide component in the aloe gel and leaf extracts are responsible or contribute to a large extent to the atenolol transport enhancement effect. It also indicates that the precipitated polysaccharides have potentially higher ability as drug absorption enhancing agents than the *A. vera* gel and whole leaf extract materials. Although not all *in vitro* transport enhancement effects may manifest as clinically significant improvements on bioavailability, the results from this study emphasises the need for future *in vivo* studies to confirm the efficacy of precipitated polysaccharides from *A. vera* materials as potential drug absorption enhancing agents.

## Conclusions

All the aloe materials investigated in this study showed the ability to reduce the TEER of excised rat intestinal tissue statistically significantly. This indicates their ability to open tight junctions between epithelial cells. In spite of this reduction in TEER, only the precipitated polysaccharide fraction from the Daltonmax700^®^ *A. vera* dehydrated gel could significantly enhance atenolol transport across excised rat intestinal tissue. In addition, the effect of the precipitated polysaccharides from the Daltonmax700^®^ *A. vera* whole leaf extract on atenolol transport was prominent when compared to the control group. The *A. ferox* materials did not increase atenolol transport compared to the control group probably due to the absence of aloverose. The results from this study therefore indicate that the polysaccharide component is responsible or contributes largely to the drug absorption enhancing effect of the *A. vera* leaf materials. Polysaccharides from aloe leaf gel material are therefore potential functional excipients to be included in novel pharmaceutical formulations to enhance drug absorption after oral administration. However, these materials should be tested *in vivo* to confirm if the bioavailability enhancement effect is clinically significant.

## Experimental

### Materials

*Aloe vera* (*Aloe barbadensis* Mill.) whole leaf extract (AVWL) and *A. vera* gel (AVG) materials were gifts from the International Aloe Science Council (IASC051309, Texas, USA). *A. vera* dehydrated gel or Daltonmax 700^®^ gel (DMG) and *A. vera* whole leaf spray dried extract or Daltonmax700^®^ whole leaf extract (DMWL) were received as donations from Improve, USA, inc. Extracted *A. ferox* polysaccharide (AFP), *A. ferox* whole leaves (AFWL) and *A. ferox* gel (AFG) fillets were received from Organic Aloe (Albertinia, South Africa). The fillets were liquidised in a food processor and then lyophilised. Polysaccharides were extracted from *A. vera* dehydrated gel or Daltonmax700^®^ gel (DMGP) and from *A. vera* spray dried whole leaf extract or Daltonmax700^®^ whole leaf extract (DMWLP) in aqueous solution by adding absolute ethanol to the volume of four times of that of the aqueous solution. The polysaccharides were separated centrifugally at 4000 r/min for 10 min and washed with 80 % v/v ethanol. The precipitate was washed 4 times after which the precipitate was separated by filtration and freeze-dried.

### Methods

#### ^1^H-NMR fingerprinting of materials

An amount of 50 mg of each of the aloe materials and precipitated polysaccharides together with 5 mg of the internal standard (nicotinic acid amide or NSA) were dissolved in 1 ml of D_2_O and their ^1^H-NMR spectra were recorded with an Avance 300HZ NMR spectrometer (Bruker).

#### Preparation of rat intestinal tissue

The study on rats was approved by the Ethics Committee of the North-West University (project number: NWU-0018-09-A5). Un-fasted adult male Sprague-Dawley rats (350–450 g) were obtained from the Laboratory Animal Centre at the Potchefstroom campus of the North-West University, South Africa. The rats were euthanized by halothane inhalation. An abdominal incision was made and a 20–30 cm intestinal segment was excised approximately 10 cm from the stomach. The intestinal segment was rinsed with ice cold Krebs-Ringer bicarbonate buffer and the serosal layer was removed by blunt dissection. The tissue was continually kept cold in ice cold Krebs-Ringer bicarbonate buffer. The excised jejunum segment was cut along the mesenteric border and cut into strips of 3 cm long. The excised jejunum segments were mounted onto the half cells of a Sweetana-Grass diffusion apparatus where after the cells were clamped together and inserted into the heating block. The buffer was circulated by a gas-lift using 95% O_2_/5% CO_2_ at a flow rate of 15–20 ml/min. The cells were kept in the heating block at 37 °C for the entire study.

#### Transepithelial electrical resistance of excised rat intestinal tissue

To be consistent with the transport studies, the test solutions contained each of the selected aloe materials respectively in Krebs-Ringer bicarbonate buffer at a concentration of 2% w/v and atenolol (20 mM) adjusted to a pH of 7.4. Atenolol alone was used as the control group and sodium lauryl sulphate (SLS) at a concentration of 0.2% w/v together with atenolol was used as the positive control group. Prior to the transepithelial electrical resistance (TEER) experiments, 7 ml Krebs-Ringer bicarbonate buffer was added to the apical and basolateral sides of the chambers for 30 min to ensure equilibrium was reached. The buffer was removed from both sides and replaced by 7 ml of the test solutions on the apical side and with 7 ml Krebs-Ringer bicarbonate buffer on the basolateral side. The TEER was measured at time intervals of 20 min for a period of 120 min in triplicate and the values obtained at time zero was used as 100%. The reversibility of the effect of the aloe materials on the TEER of the excised rat intestinal mucosal tissue was measured by removing the test solutions and buffer from the apical and basolateral sides after 120 min and replacing them with 7 ml Krebs-Ringer bicarbonate buffer on both sides. The TEER was then measured for a further 60 min after removal of the test solutions at time intervals of 20 min. All experiments were done in triplicate at 37 °C.

#### Atenolol transport across excised rat intestinal tissue

Transport of atenolol in the apical-to-basolateral direction was determined in the absence (normal control) and presence of the selected aloe materials at a concentration of 2% w/v in Krebs-Ringer bicarbonate buffer adjusted to a pH of 7.4. Sodium lauryl sulphate (SLS) at a concentration of 0.2% w/v together with atenolol was used as a positive control. SLS is a known intestinal absorption enhancer that exerts its effect by opening of tight junctions [21]. The concentration of atenolol used throughout the study was 20 mM. Prior to the transport experiments, 7 ml Krebs-Ringer bicarbonate buffer (pH 7.4) was added to the apical and basolateral chambers of the diffusion chamber for 30 min to make sure equilibrium was reached. After this equilibration period, the buffer was removed from both sides and replaced with 7 ml test solution on the apical side and 7 ml Krebs-Ringer bicarbonate buffer on the basolateral side of the intestinal membrane. Samples of 200 μl were taken at 20, 40, 60, 80, 100 and 120 min from the basolateral side. The samples withdrawn from the basolateral side were replaced with an equal volume of Krebs-Ringer bicarbonate buffer. All the experiments were done in triplicate at 37 °C at a pH of 7.4. The TEER was measured before and after the study to ensure the integrity of the membrane was maintained throughout the transport study.

Atenolol was quantified by means of reversed-phase high-performance liquid chromategraphy (RP-HPLC). The HPLC system consisted of a Waters 2690 separation module and a Waters 996 photodiode array detector (Waters, MA, USA). Separation was achieved on a Phenomenex C_18_ column (250 mm × 4.6 mm; 5μm, USA) equipped with a C_18_ guard column (Phenomenex, USA) and operated at room temperature. The chromatographic data was collected and analysed using Empower software. The mobile phase consisted of (A) 0.1 M acetic acid; (B) methanol at a flow rate of 1.0 ml/min; a gradient elution was obtained as follows: 85% A: 15% B to 70% A: 30% B in 10 min and changed to 85% A: 15% B in 1 min. Atenolol was detected at a wavelength of 273 nm. The injection volume was 20 μl. Since a validated analytical method was used, only linearity was determined.

#### Data analysis

The atenolol concentrations in the transport samples were corrected for dilution and plotted as percentage cumulative atenolol transport as a function of time. The apparent permeability coefficients (P_app_) were calculated using the following equation [19]:

$${\text{P}}_{\text{app}}=\left(\frac{\text{dc}}{\text{dt}}\right)\left(\frac{1}{\text{A}\times 60\times {\text{C}}_{0}}\right)$$

Where dc/dt is the amount of atenolol transported within a given time period, A is the surface area of the insert, and C_0_ is the initial drug concentration.

Permeation-enhancement ratios (R) were calculated with the following equation [19]:

$$\text{R}=\frac{{\text{P}}_{\text{app}}\;\text{test}}{{\text{P}}_{\text{app}}\;\text{control}}$$

Where R is the permeation-enhancement ratio, P_app_ test is the apparent permeability coefficient value of the test solution and P_app_ control is the apparent permeability coefficient value of the control solution.

The results were statistically analyzed by means of one-way repeated analysis of variance (ANOVA) to determine the differences between TEER values and apparent permeability coefficients. Differences were considered statistically significant if *P* < 0.05.

## Figures and Tables

**Tab. 1.** Composition of the *A. vera* gel and whole leaf materials as well as precipitated polysaccharides as determined by ^1^H-NMR spectroscopy

**Compound**	**Content [%]**					
	**AVG**	**AVWL**	**DMG**	**DMWL**	**DMGP**	**DMWLP**
Aloverose	12.7	5.5	15.2	4,9	8.0	25.0
Glucose	16.7	Detected	9.8	8,6	Trace	ND
Malic acid	20.0	1.2	20.7	24.7	18.8	9.0
Lactic acid	5.1	21.5	ND	ND	ND	ND
Citric acid	ND	16.9	2.0	8.9	15.3	ND
WLM	Detected	11.4	ND	14.6	15.7	ND
Maltodextrin	ND	ND	ND	ND	ND	ND
Acetic acid	ND	0.6	ND	ND	ND	ND
Succinic acid	Trace	Detected	ND	Detected	Detected	ND

AVG = *Aloe vera* gel; AVWL = *Aloe vera* whole leaf; DMG = Daltonmax700^®^ gel; DMWL = Daltonmax700^®^ whole leaf; DMGP = Daltonmax700^®^ gel precipitated polysaccharides; DMWLP = Daltonmax700^®^ whole leaf precipitated polysaccharides; WLM = whole leaf marker; ND = not detected.

**Tab 2.** Apparent permeability coefficient (P_app_) values and transport-enhancement ratio (R) values for atenolol transport across excised rat intestinal tissue in the absence and presence of the selected aloe materials

**Experimental group**	**P_app_ × 10^–6^ (cm/s) at pH 7.4**	**R**
AFG	0.81 ± 0.07	0.70
AFP	0.84 ± 0.32	0.73
AFWL	0.78 ± 0.03	0.67
AVG	1.24 ± 0.10	1.07
AVWL	0.81 ± 0.23	0.70
DMG	0.94 ± 0.34	0.81
DMGP	2.14 ± 0.33^#^	1.84
DMWL	0.74 ± 0.18	0.64
DMWLP	2.06 ± 0.73	1.78
Control (atenolol alone)	1.16 ± 0.27	1.00
Positive control (SLS)	2.13 ± 0.06^#^	1.84

AVG = *Aloe vera* gel; AVWL = *Aloe vera* whole leaf; AFG = *Aloe ferox* gel;

AFWL = *Aloe ferox* whole leaf; AFP = *Aloe ferox* polysaccharide;

DMG = Daltonmax700^®^ gel; DMWL = Daltonmax700^®^ whole leaf;

DMGP = Daltonmax700^®^ gel precipitated polysaccharides;

DMWLP = Daltonmax700^®^ whole leaf precipitated polysaccharides.

# Statistically significantly different from the control group (atenolol alone).
